# Case Report: Aortoesophageal Fistula induced by a fish bone: the critical role of mediastinal infection control after TEVAR and endoscopic closure

**DOI:** 10.3389/fcvm.2025.1722954

**Published:** 2026-01-15

**Authors:** Yue Ma, Lin Zhang, Fucheng Ji

**Affiliations:** 1 Department of Anesthesiology, Qilu Hospital (Qingdao), Cheeloo College of Medicine, Shandong University, Qingdao, Shandong, China; 2 Department of Anesthesia and Perioperative Medicine, Qingdao Central Hospital, University of Health and Rehabilitation Sciences, Qingdao, Shandong, China

**Keywords:** aortoesophageal fistula, endoscopic treatment, infection, thoracic endovascular aortic repair, upper gastrointestinal bleeding

## Abstract

**Background:**

Foreign-body–related aortoesophageal fistula (AEF) is rare and fatal without rapid hemostasis and infection control.

**Case:**

A 74-year-old woman presented with hematemesis and shock after suspected fish-bone ingestion. Emergency TEVAR with a 32 mm covered stent-graft (proximal landing in the distal third of the left subclavian artery) controlled bleeding. Endoscopic clip-and-line closure of an approximately 15 mm esophageal defect was performed. Despite broad-spectrum antimicrobial therapy, the patient developed recurrent fever and later rebleeding associated with mediastinal sepsis.

**Conclusion:**

TEVAR and endoscopic repair require an integrated, early source-control strategy. A stepwise algorithm prioritizing timely mediastinal drainage is essential to prevent graft contamination, infectious relapse, and rebleeding.

## Introduction

An aortoesophageal fistula (AEF) is a rare yet highly fatal condition, often leading to rapid patient demise from acute hemorrhagic shock or severe sepsis (1). The cornerstone of emergency management involves thoracic endovascular aortic repair (TEVAR) to control aortic bleeding, combined with endoscopic closure of the esophageal fistula and anti-infection therapy, aiming to preserve the integrity of both the aorta and esophagus. This report presents a case of a delayed thoracic AEF caused by a fish bone. Although initial anatomical repair was achieved via TEVAR and endoscopic clipping, persistent mediastinal infection ultimately led to recurrent hematemesis due to inadequate infection control. This case underscores that while the combination of TEVAR and endoscopy can rapidly control bleeding and repair the fistula, failure to effectively manage mediastinal infection may lead to treatment failure, highlighting the critical role of infection control within the comprehensive treatment strategy.

## Case presentation

**Figure 1:**
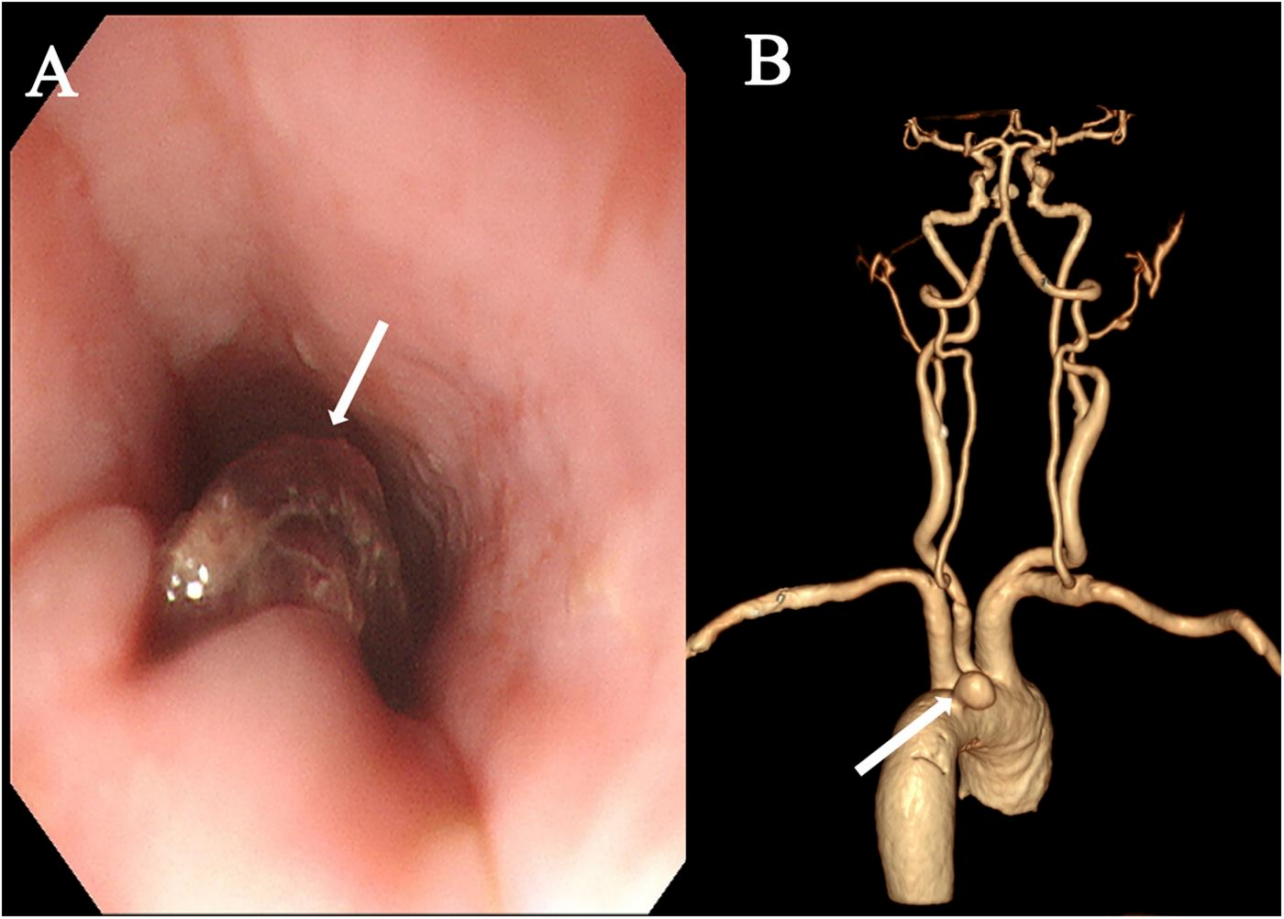
Preoperative examination. **(A)** Emergency gastroscopy revealing a suspected fish bone foreign body within the esophagus (arrow). **(B)** Thoracic aortic CTA demonstrated a ruptured pseudoaneurysm (arrow).

A 74-year-old female was admitted with a 10-day history of chest pain radiating to the back after eating fish, and a 6 h history of hematemesis (approximately 300 ml). An emergency gastroscopy at a local hospital revealed a foreign body (suspected fish bone) 20 cm from the incisors (Figure 1A). Upon transfer to our emergency room, she experienced another episode of massive hematemesis. On admission, she was lethargic with a heart rate of 140 beat per minute, blood pressure of 81/43 mmHg, SpO_2_ of 75%, hemoglobin of 43 g/L, and lactate of 7.2 mmol/L. Emergency endotracheal intubation was performed. A thoracic aortic computed tomography angiography (CTA) demonstrated a ruptured pseudoaneurysm (Figure 1B). From the patient's viewpoint, the initial hemorrhage was abrupt and terrifying. She valued rapid bleeding control but later felt anxious due to recurrent fever and the possibility of rebleeding. Clear communication about infection control steps and follow-up planning was essential to her sense of safety.

**Figure 2:**
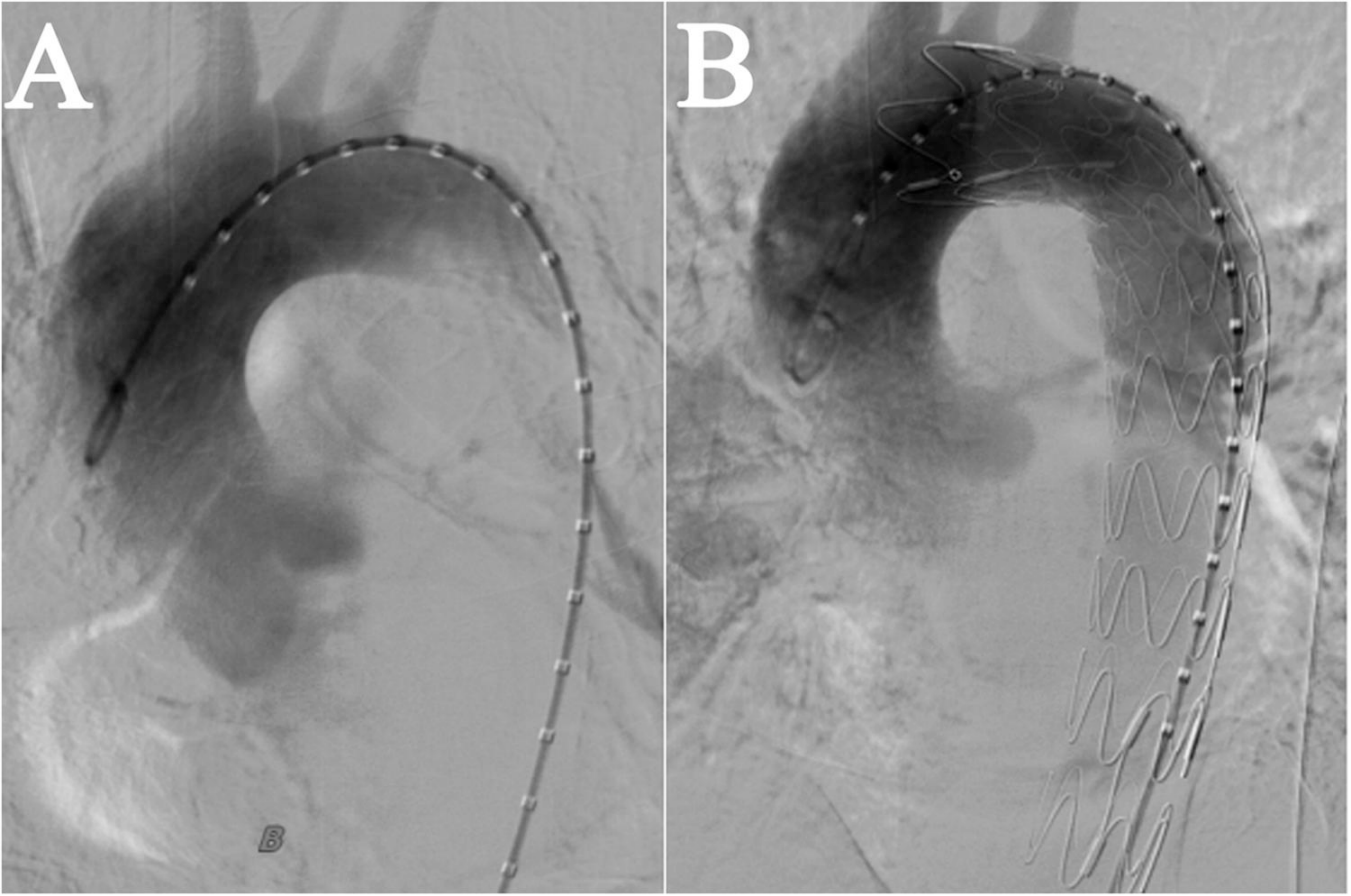
Intraoperative imaging in TEVAR. **(A)** Prior to Stent Implantation. **(B)** After stent implantation, blood flow in the left subclavian artery was normal, with no evidence of leakage from the aorta.

We performed an emergency TEVAR procedure on the patient under general anesthesia. The angiogram revealed a localized rupture in the thoracic aorta located approximately 6 mm distal to the left subclavian artery, with evident massive contrast extravasation. The proximal end of the stent graft was positioned in the distal third of the left subclavian artery. The deployment proceeded smoothly, and the stent graft achieved a satisfactory morphological appearance. A subsequent aortic angiogram confirmed that the thoracic stent graft had good wall apposition, with no evidence of endoleak, and the aortic rupture was successfully excluded. Blood flow to the left subclavian artery remained well preserved, obviating the need for a carotid-subclavian bypass procedure (Figure 2). The spinal cord protection strategy employed throughout the procedure involved maintaining the mean arterial pressure above 65 mmHg.

**Figure 3:**
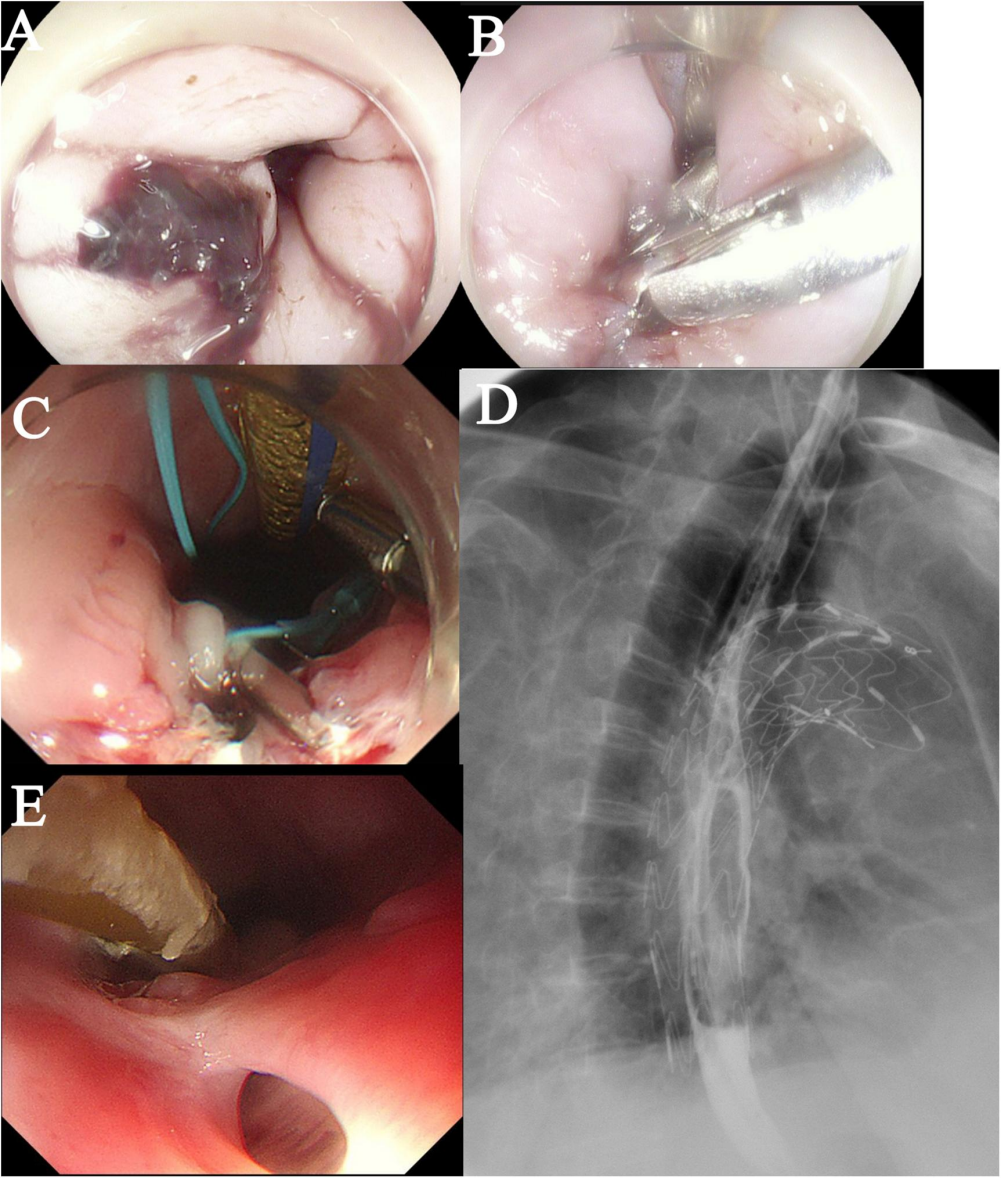
Postoperative esophageal evaluation. **(A)** A bleeding laceration (approximately 1.5 cm in length) is observed in the esophagus. **(B)** The defect was closed using endoscopic clipping. **(C)** The laceration was re-closed with three titanium clips reinforced with a nylon loop. **(D)** A barium esophagogram confirmed good esophageal integrity with no evidence of leakage. **(E)** A follow-up endoscopy showed an intact external esophageal wall.

On Postoperative Day 2, gastroscopy revealed a 15 mm laceration in the esophagus approximately 20 cm from the incisors (Figure 3A). Given the patient's frail condition and financial constraints, the defect was closed using two titanium clips (Figure 3B). On Day 8, a repeat gastroscopy showed that the initial clips had dislodged. After debridement of the site, the laceration was re-closed with three titanium clips reinforced with a nylon loop (Figure 3C). Enteral nutrition via a nasojejunal tube was initiated on Day 9, with strict instructions to maintain nil-by-mouth status.

Empirical antibiotic therapy with cefoperazone-sulbactam plus tinidazole was administered from Day 0 to Day 4. During this period, the patient's body temperature(T) fluctuated between 37.0°C and 37.5 °C, and the procalcitonin (PCT) level decreased from 6.202 ng/mL to 1.202 ng/mL (Normal value <0.05 ng/mL). The regimen was then escalated to piperacillin-tazobactam, after which the body temperature dropped below 37.0°C and the PCT level continued to decline. On Day 9, sputum culture identified ESBL-producing bacteria and Acinetobacter baumannii. Accordingly, the antibiotic therapy was changed to meropenem, and tinidazole was discontinued. On Day 16, sputum culture grew Pseudomonas aeruginosa, leading to the addition of moxifloxacin to the regimen, which was continued until discharge. A barium esophagogram performed on Day 15 confirmed good esophageal integrity with no evidence of leakage (Figure 3D). The patient was discharged on Day 29 in a stable condition (T:36.6°C, PCT: 0.022 ng/mL) and a chest CT showing no significant mediastinal infection.

The patient was discharged with instructions to continue anti-infective therapy at a local hospital and to avoid oral intake. On Day 40, a follow-up endoscopy at our hospital showed an intact external esophageal wall (Figure 3E). However, a sinus tract was found, debrided, and closed again with three titanium clips. The patient was advised to commence a liquid diet orally.

**Figure 4:**
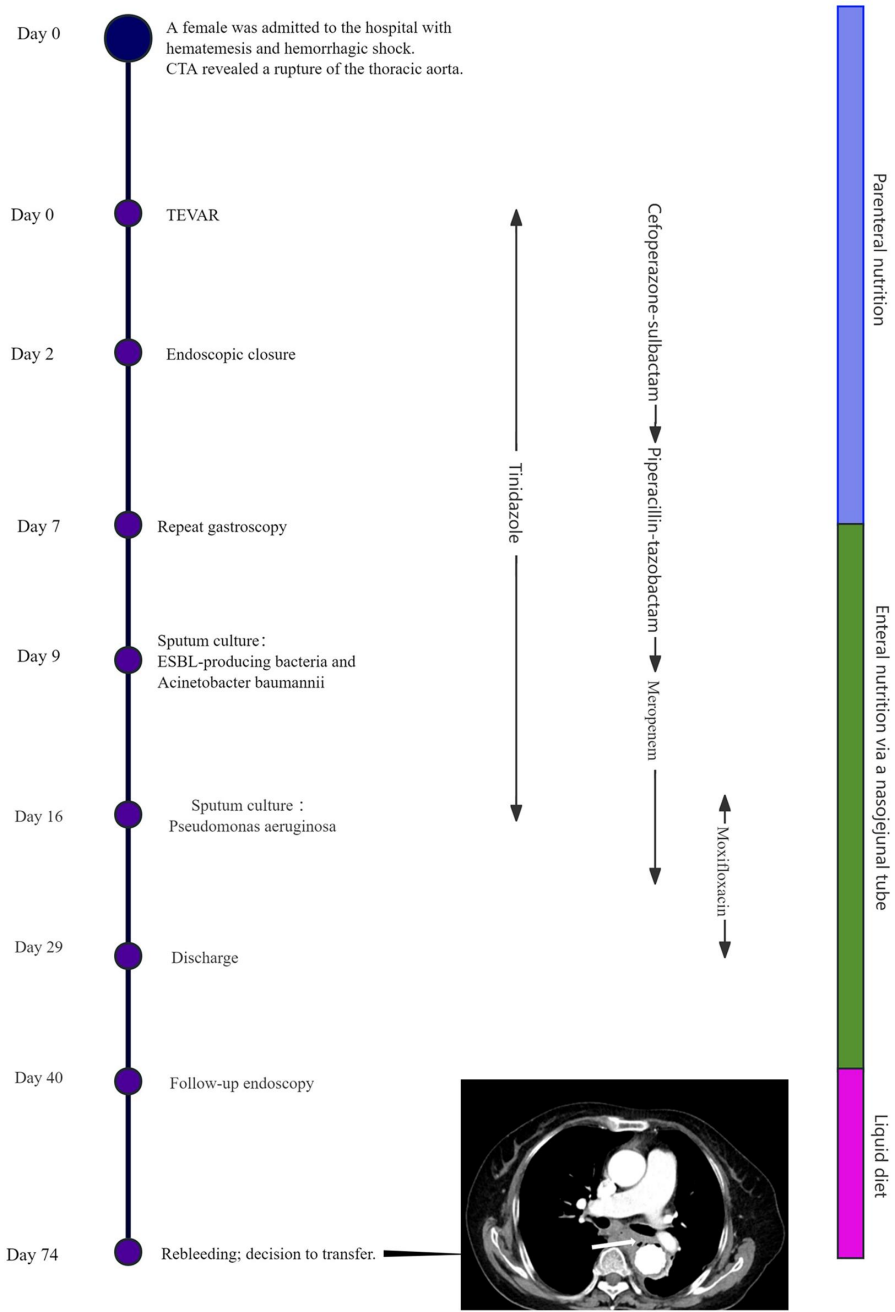
Timeline of treatment. Chest CT revealed the presence of gas adjacent to the aorta, suggestive of mediastinal infection (arrow).

Unfortunately, on Day 74, the patient was readmitted with hematemesis and fever. A chest CT revealed mediastinal infection (Figure 4). The medical team explicitly recommended emergency surgical exploration to perform mediastinal debridement and drainage and to assess the graft status. However, after fully understanding the necessity and risks of the procedure—including the extremely high mortality rate—the patient and her family ultimately declined all further invasive interventions. It was later learned that the patient passed away several days after discharge. The treatment timeline is depicted in Figure 4.

## Discussion

AEF is a rare and lethal condition, with common etiologies including thoracic aortic aneurysm, esophageal foreign bodies, and thoracic malignancies (2). Its classic presentation can be Chiari's triad (chest pain, sentinel hematemesis, and fatal hemorrhage) (3). The primary treatment goals involve controlling acute bleeding, maintaining the continuity of the aorta and esophagus, and preventing/treating infection. While traditional open surgery can be life-saving, it carries a high mortality rate (reported up to 45.4%–55%) (4). In contrast, TEVAR, being rapid, minimally invasive, and cost-effective, is increasingly utilized. However, AEF is often complicated by infection, and patients are frequently debilitated. A consensus on the optimal method for effective esophageal fistula closure remains elusive.

This case illustrates the management of a patient with AEF and hemorrhagic shock caused by a fish bone, involving emergency airway management, TEVAR for hemorrhage control, and endoscopic fistula closure. Although systemic anti-infective therapy initially normalized infection markers and resolved symptoms, the patient experienced intermittent fever after discharge, culminating in recurrent hematemesis. This outcome indicates that for elderly and frail patients, while the combination of TEVAR and endoscopy offers a minimally invasive option, failure to achieve adequate control of mediastinal infection can lead to treatment failure, emphasizing the paramount importance of comprehensive infection management.

Emergency TEVAR is an effective measure for controlling acute massive bleeding in AEF (5). However, some studies associate TEVAR with higher mortality, potentially because patients selected for TEVAR are often older, frail, and high-risk candidates unsuitable for open surgery (6). Nevertheless, comparisons of postoperative complications between open surgical repair (OSR) and TEVAR have not shown significant differences in adverse event rates (6). In patients with severe concomitant mediastinal infection, unsuccessful infection control post-TEVAR may progress to sepsis, increasing mortality risk. Successful infection control via Video-Assisted Thoracoscopic Surgery (VATS) for mediastinal drainage has been reported (7). In our case, within this critical context of rapid exsanguination, the primary and most immediate life-saving goal was to achieve definitive control of the aortic rupture. Therefore, we proceeded directly with emergency TEVAR as the definitive hemostatic intervention. We determined that alternative measures, such as endoscopic interventions or balloon tamponade, would have been unlikely to secure control of the bleeding source originating from the high-pressure aortic system and could have delayed the critical repair, potentially exacerbating the situation. Considering the patient's advanced age, frailty, severe anemia, and no significant evident of mediastinal infection on post-TEVAR CT, an initial non-surgical approach combining endoscopic therapy and antibiotics was selected.

Managing the esophageal fistula after TEVAR is critical, as improper repair carries risks of AEF recurrence or stent infection. Strategies to restore esophageal integrity include open esophageal resection, endoscopic therapies, or expectation for spontaneous healing (8). Czerny and colleagues suggest that open surgery can effectively address refractory mediastinal infection (9), but many patients opt for conservative treatment due to economic factors or the significant trauma of surgery. With advancements in endoscopic techniques, their use for fistula closure has increased (3, 10, 11), although efficacy can be uncertain. Larger fistulas warrant active intervention to avoid complications from prolonged nil-by-mouth status. Esophageal stenting, while an option for fistula closure, has not been proven to reduce mortality and carries risks of migration and infection (12). Spontaneous healing of small fistulas without endoscopic intervention has been reported, albeit over a prolonged period (e.g., 15 months) (13). Our case employed titanium clip closure, achieving initial isolation but not complete mucosal healing. Reports on the combined use of TEVAR and endoscopic closure for AEF are relatively scarce, and long-term outcome data are lacking. This highlights the critical importance of radical surgical debridement in achieving definitive source control. The combined approach we described, which integrates endovascular stabilization and efforts to control the esophageal leak, may be considered a potentially viable alternative for a specific subset of high-risk patients who are unsuitable for major surgery.

Controlling mediastinal infection is as crucial as repairing the vascular and esophageal defects. Not all infections are manageable conservatively. If antibiotic therapy is ineffective, VATS drainage or even open surgery may be necessary (7). According to the literature, not all cases of AEF caused by foreign bodies require mediastinal drainage. The decision to pursue conservative management or concurrent VATS for mediastinal drainage should be based on the specific nature of the foreign body. If the foreign body is considered “clean” (i.e., posing a low risk of causing severe infection), initial treatment with intravenous antibiotics may be sufficient. Postoperatively, patients should be closely monitored for signs of infection, including body temperature, white blood cell count, and PCT levels. The persistence of fever, failure of PCT to decrease, or a recurrent rise in PCT should raise strong suspicion for a persistent or new mediastinal infection. This clinical assessment must be combined with serial chest CT scans. The emergence of new or expanding hydropneumatic collections, abscess formation, or the presence of gas shadows around the stent graft on CT are objective indicators warranting intervention, irrespective of the patient's clinical presentation at that moment.

The primary goal immediately postoperatively was to address the high probability of a polymicrobial infection involving both aerobic and anaerobic bacteria, which are commonly associated with oropharyngeal and gastrointestinal flora in the context of suspected AEF. Cefoperazone-sulbactam was selected to provide extended coverage against a wide range of Gram-positive and Gram-negative bacteria, while tinidazole was added specifically to ensure reliable activity against anaerobic organisms frequently implicated in such infections. This combination aligns with the principle of initiating broad-spectrum therapy for life-threatening infections prior to pathogen identification, aiming to rapidly control the overwhelming infection and sepsis. A favorable initial response was observed, evidenced by a reduction in both body temperature and PCT levels. However, subsequent microbiological findings, including sputum culture, revealed drug-resistant bacteria, necessitating an escalation to a broader-spectrum antibiotic regimen. In retrospect, given the severe consequences of AEF-associated mediastinal infection and potential stent graft infection, initiating empiric therapy with a broader-spectrum, more potent antibiotic from the outset might have potentially avoided the subsequent need for prolonged antibiotic courses and the emergence of resistant strains that complicated infection control.

The patient's follow-up endoscopic examination after discharge revealed an intact external esophageal wall. A liquid diet was initiated following simple clip closure, but this timing may have been premature. Delaying oral intake until complete esophageal closure is confirmed could potentially reduce the risk of recurrent infection (13). Additionally, although the patient reported adhering to our antibiotic regimen at a local hospital, the specific details of the post-discharge anti-infective protocol remain unclear and may be associated with the recurrence. Therefore, for patients who are candidates for conservative management, initiating broad-spectrum antibiotic therapy from the outset, along with delaying oral intake, may help reduce the potential for mediastinal infection. Furthermore, strict monitoring of vital signs, infection biomarkers, and radiographic findings is essential, with surgical mediastinal drainage performed when necessary.

Foreign-body-induced AEF primarily results from direct mechanical penetration by an ingested sharp object, often leading to a more localized defect. In contrast, AEF caused by malignancy typically arises from tumor erosion into the aortic wall, while iatrogenic cases are frequently associated with complications from procedures like TEVAR or esophageal surgery (14). The therapeutic goals also differ significantly: management of foreign-body AEF aims for curative repair through a combined approach (e.g., TEVAR for aortic control plus surgical drainage and esophageal closure), whereas treatment for malignancy-associated AEF is often palliative, focusing on hemorrhage control and infection management due to the underlying advanced disease. Consequently, survival outcomes are generally more favorable in carefully selected foreign-body AEF cases where definitive source control is achievable.

## Conclusion

In conclusion, effective long-term management of foreign body-induced AEF requires rigorous prevention and control of mediastinal infection beyond initial TEVAR. Treatment should be individualized. When active infection is absent, a comprehensive postoperative regimen—including targeted broad-spectrum antibiotics, delayed oral intake until endoscopic confirmation of closure, and close monitoring—is essential. For patients with established infection, a single-stage hybrid approach combining TEVAR with surgical drainage is crucial for definitive source control. This tailored, multifaceted strategy may significantly reduce devastating infective complications and improve outcomes in this highly lethal condition.

## Data Availability

The datasets presented in this study can be found in online repositories. The names of the repository/repositories and accession number(s) can be found in the article/Supplementary Material.
